# The association between intraocular pressure dynamics during dark-room prone testing and intraocular pressure over a relatively long-term follow-up period in primary open-glaucoma patients

**DOI:** 10.1007/s00417-023-06257-0

**Published:** 2023-10-21

**Authors:** Masataka Sato, Naoki Kiyota, Takeshi Yabana, Shigeto Maekawa, Satoru Tsuda, Kazuko Omodaka, Noriko Himori, Yu yokoyama, Toru Nakazawa

**Affiliations:** 1 Department of Ophthalmology, Tohoku University Graduate School of Medicine, Miyagi, Japan; 2 Department of Aging Vision Healthcare, Tohoku University Graduate School of Biomedical Engineering, Miyagi, Japan; 3 Department of Ophthalmic Imaging and Information Analytics, Tohoku University Graduate School of Medicine, Miyagi, Japan; 4 Department of Retinal Disease Control, Tohoku University Graduate School of Medicine, Miyagi, Japan; 5 Department of Advanced Ophthalmic Medicine, Tohoku University Graduate School of Medicine, Miyagi, Japan

**Keywords:** Glaucoma, Dark-room prone testing, Intraocular pressure fluctuation, Maximum intraocular pressure

## Abstract

**Purpose:**

To investigate the relationship between the dynamics of intraocular pressure (IOP) during dark-room prone testing (DRPT) and IOP over a relatively long-term follow-up period.

**Methods:**

This retrospective study enrolled 84 eyes of 51 primary open-angle glaucoma patients who underwent DRPT for whom at least three IOP measurements made using Goldmann applanation tonometry were available over a maximum follow-up period of two years. We excluded eyes with a history of intraocular surgery or laser treatment and those with changes in topical anti-glaucoma medication during the follow-up period. In DRPT, IOP was measured in the sitting position, and after 60 min in the prone position in a dark room, IOP was measured again. In this study, IOP fluctuation refers to the standard deviation (SD) of IOP, and IOP max indicates the maximum value of IOP during the follow-up. The relationship between these parameters was analyzed with a linear mixed-effects model, adjusting for clinical parameters including age, gender, and axial length.

**Results:**

IOP increased after DRPT with a mean of 6.13 ± 3.55 mmHg. IOP max was significantly associated with IOP after DRPT (β = 0.38; *p* < 0.001). IOP fluctuation was significantly associated with IOP change in DRPT (β = 0.29; *p* = 0.007).

**Conclusion:**

Our findings suggest that short-term and relatively long-term IOP dynamics are associated. Long-term IOP dynamics can be predicted by DRPT to some extent.


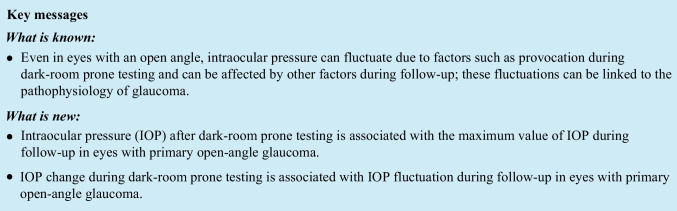


## Introduction

Glaucoma is an ocular neurodegenerative disorder characterized by the progressive loss of retinal ganglion cells and corresponding visual field (VF) defects [1]. While numerous factors are implicated in the pathophysiology of glaucoma [2], lowering intraocular pressure (IOP) remains the major evidence-based treatment for this condition [3]. However, IOP is subject to fluctuations due to various factors, such as dietary habits, exercise, seasonal variations, and angle status [4–6]. Importantly, it has been reported that maximum IOP and the degree of IOP fluctuation during follow-up are associated with glaucoma progression [7–13]. Consequently, it is crucial to perform multiple IOP measurements to evaluate IOP dynamics rather than rely on a single assessment. This requires relatively long-term monitoring (spanning several months or years), which can be time-consuming and may prevent ophthalmologists from making rapid decisions regarding treatment plans.

Conversely, postural alterations can be employed to examine IOP dynamics over a relatively short period of time (ranging from several minutes to an hour) [14, 15]. The most representative test of postural changes is dark-room prone testing (DRPT), initially devised to identify angle-closure glaucoma [16]. Nonetheless, previous studies have shown that IOP increases in the prone position, even in open-angle eyes, suggesting that elevated IOP may not be exclusive to angle-closure cases [17].

Long-term IOP fluctuations and short-term IOP changes due to postural change may be closely linked in eyes with primary open-angle glaucoma (POAG), potentially contributing to its pathophysiology. However, the relationship between these two types of IOP dynamics in eyes with POAG remains poorly understood. In light of this, the current study aims to examine the relationship between IOP dynamics during DRPT and IOP during long-term follow-up in patients with POAG.

## Materials and methods

**Fig. 1:**
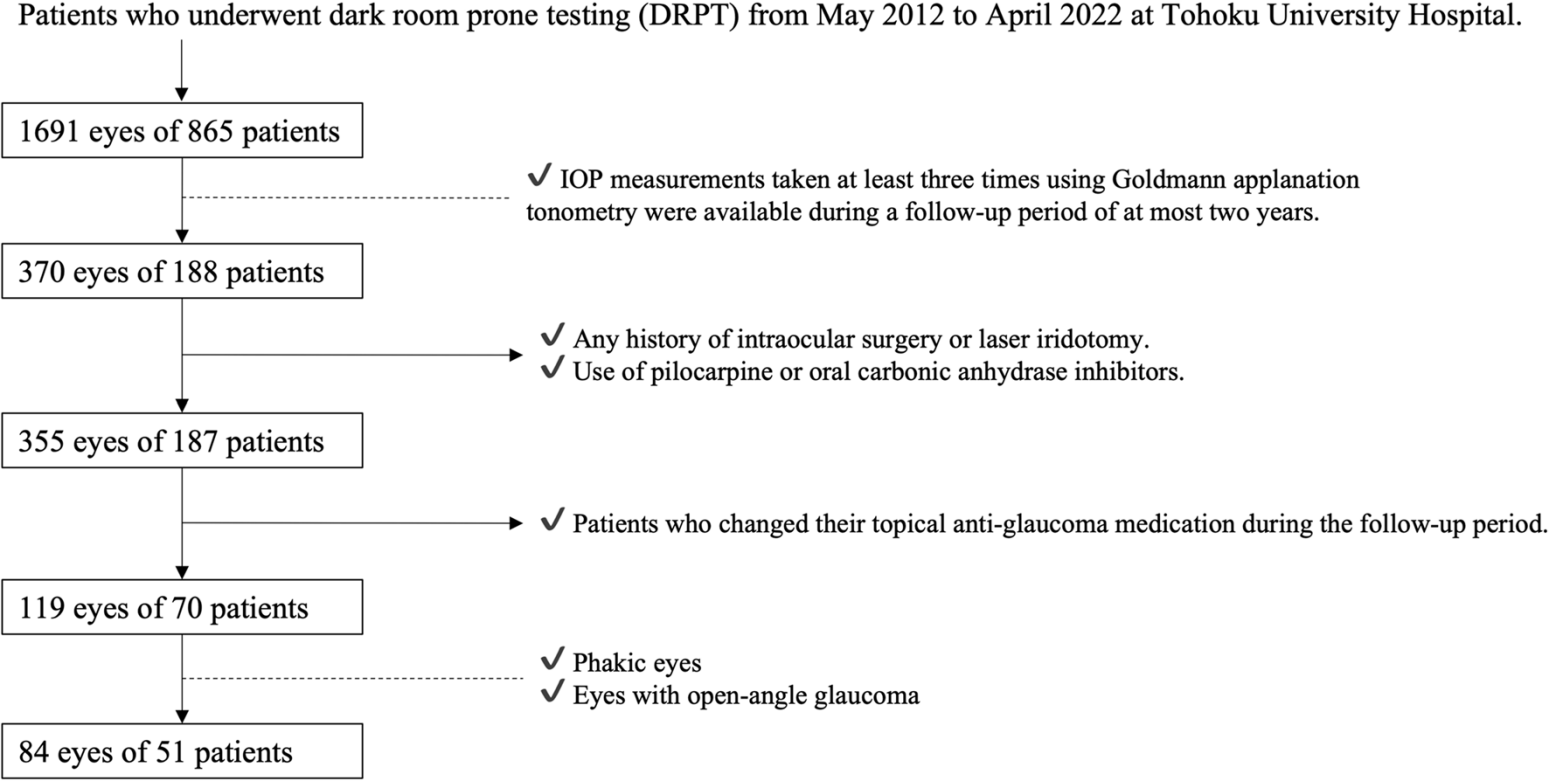
Selection schema for eyes included in this study. The dotted lines indicate the inclusion criteria, and the horizontal arrows indicate the exclusion criteria

This retrospective study enrolled 84 eyes of 51 POAG patients (male to female ratio = 35:16) who attended Tohoku University Hospital, situated in Miyagi, Japan, between August 2014 and November 2021. Written informed consent was acquired from all participants. The study adhered to the principles outlined in the Declaration of Helsinki and received approval from the Ethics Committee of Tohoku University School of Medicine (protocol number: 2021–1-430). The diagnosis of POAG was made by a glaucoma specialist (T.N.) and was based on characteristics that included an open angle in a gonioscopic examination, the presence of glaucomatous optic nerve head changes and corresponding VF defects matching the Anderson–Patella criteria [18], and the absence of other diseases that can affect the VF. We excluded secondary open-angle glaucoma patients because IOP can fluctuate in these patients due to the cause of the disease [19, 20]. Additional inclusion criteria for this study were (1) availability of at least three IOP measurements using Goldmann applanation tonometry (GAT) during follow-up and (2) that the eye was phakic. The exclusion criteria were (1) any history of intraocular surgery or laser iridotomy, (2) use of pilocarpine or oral carbonic anhydrase inhibitors, and (3) changes in the use of topical anti-glaucoma medication during the follow-up period. A flowchart illustrating the selection process is presented in Fig. 1. In cases where both eyes of a patient fulfilled the inclusion criteria, both eyes were incorporated into the statistical analysis.

Axial length (AL) was measured with the IOL Master (Zeiss Meditec, Dublin, CA, USA); retinal nerve fiber layer thickness was measured with swept-source optical coherence tomography (OCT; DRI OCT, Triton, Topcon, Inc., Tokyo, Japan); the VF was measured with the SITA standard 24–2 program of the Humphrey Field Analyzer (Carl Zeiss Meditec, Dublin, CA); and only measurements with fixation errors < 20%, false positives < 33%, and false negatives < 33% were used, following our previous reports [21, 22].

**Fig. 2:**
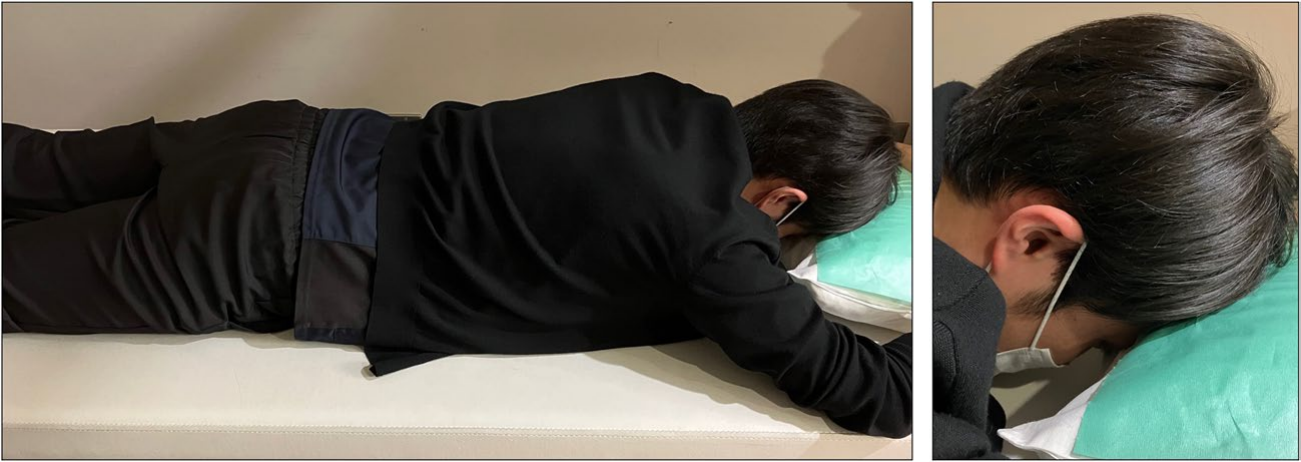
Representative photos of a subject undergoing dark-room prone testing. Note that the representative images were captured in a lighted room for better visibility. The overall view is displayed on the left, with a close-up of the head featured on the right. Patients are positioned prone on the bed with their heads resting on a pillow. An experienced examiner ensures that no pressure is applied to the eyeballs and that patients do not fall asleep during the test

All IOP measurements, including DRPT, were done with GAT. IOP baseline during DRPT was measured in the sitting position. As shown in Fig. 2, after 60 min in the prone position in a dark room [16, 17], IOP was again measured with GAT in the sitting position (i.e., IOP after DRPT). IOP change during DRPT indicates the increment in the value of IOP after DRPT compared to DRPT baseline IOP.

To mitigate the influence of changes in ocular status during the follow-up period (e.g., angle narrowing due to cataract progression) on our results, we restricted the maximum follow-up duration to two years. The term “IOP fluctuation” during the follow-up refers to the standard deviation (SD) of IOP, while “IOP max” denotes the highest IOP value recorded during this period. The term “number of eyedrops” in this study indicates the number of component drugs in anti-glaucoma eyedrops.

All data are presented as the mean ± SD. A multivariable linear mixed-effects model was employed, setting the “subject” variable as a random effect [22–24], to evaluate the association of IOP dynamics during the follow-up term and DRPT with other variables while adjusting for potential confounding factors, including age, gender, DRPT baseline IOP, axial length, and the number of eyedrops. All statistical analyses were conducted using R software version 4.1.1 (R Core Team 2021). The threshold for statistical significance was set at *p* < 0.05.

## Results

Among 1691 eyes of 865 patients who underwent DRPT from May 2012 to April 2022 at Tohoku University Hospital, 84 eyes of 51 patients met the criteria in this study.

The systemic and ocular characteristics of the POAG patients enrolled in this study are shown in Table 1. DRPT baseline IOP was 12.65 ± 2.18 mmHg. IOP increased after DRPT by 6.13 ± 3.55 mmHg. The follow-up period was 548.00 ± 181.15 days and IOP was measured 5.76 ± 2.53 times. IOP fluctuation and IOP max were 1.38 ± 0.75 mmHg and 15.38 ± 2.62 mmHg, respectively.

**Table 1:** Clinical characteristics of open-angle glaucoma patients

Systemic characteristics	
Number of patients, *n*	51
Age, years	62.55 ± 10.68
Male to female ratio, *n*	35:16
Ocular characteristics	
Number of eyes, *n*	84
DRPT baseline IOP, mmHg	12.65 ± 2.18
IOP after DRPT, mmHg	18.79 ± 4.02
IOP change during DRPT, mmHg	6.13 ± 3.55
IOP follow-up period, days	548.00 ± 181.15
IOP measurement times, *n*	5.76 ± 2.53
IOP max, mmHg	15.38 ± 2.62
IOP min, mmHg	11.46 ± 2.19
IOP fluctuation, mmHg	1.38 ± 0.75
Axial length, mm	25.94 ± 1.71
Number of eyedrops, *n*	3.37 ± 1.40

*IOP* intraocular pressure, *DRPT* dark-room prone testing

IOP max/min indicate the maximum/minimum value of IOP during the follow-up

IOP fluctuation indicate the standard deviation of IOP during the follow-up

Data are expressed as mean ± standard deviation

Table 2 shows the effect of clinical factors on IOP max in a multivariable linear mixed-effects model. IOP max was significantly associated with IOP after DRPT (β = 0.38; *p* < 0.001) in a multivariable linear mixed-effects model, while age (β = -0.08; *p* = 0.551), male gender (β = 0.03; *p* = 0.817), axial length (β = -0.07; *p* = 0.548), and the number of eyedrops (β = 0.02; *p* = 0.869) did not show significant associations.

**Table 2:** The effect of clinical factors on IOP max in a multivariable linear mixed-effects model

Response variable	Explanatory variable	β	*P* value
IOP max			
	Age	-0.08	0.551
	Male gender (reference, female)	0.03	0.817
	IOP after DRPT	0.38	< 0.001*
	Axial length	-0.07	0.548
	Number of eyedrops	0.02	0.869

*DRPT* dark-room prone testing, *IOP* intraocular pressure

IOP max indicate the maximum value of IOP during follow-up

β indicates the standardized coefficient and the asterisk indicates statistical significance

Table 3 shows the effect of clinical factors on IOP fluctuation in a multivariable linear mixed-effects model. IOP fluctuation was significantly associated with IOP change during DRPT (β = 0.29; *p* = 0.007) in a multivariable linear mixed-effects model, while DRPT baseline IOP (β = 0.21; *p* = 0.062) was not significantly associated with IOP fluctuation. Similarly, age (β = 0.05; *p* = 0.687), male gender (β = 0.19; *p* = 0.148), axial length (β = -0.16; *p* = 0.204), and the number of eyedrops (β = 0.11; *p* = 0.335) did not show significant associations.

**Table 3:** The effect of clinical factors on IOP fluctuation in a multivariable linear mixed-effects model

Response variable	Explanatory variable	β	*P* value
IOP fluctuation			
	Age	0.05	0.687
	Male gender (reference, female)	0.19	0.148
	DRPT baseline IOP	0.21	0.062
	IOP change during DRPT	0.29	0.007*
	Axial length	-0.16	0.204
	Number of eyedrops	0.11	0.335
	Number of eyedrops	0.02	0.869

*DRPT*  dark-room prone testing, *IOP*  intraocular pressure

IOP fluctuation indicates the standard deviation of IOP during the follow-up

β indicates the standardized coefficient and the asterisk indicates statistical significance

## Discussion

In the current study, we investigated the relationship between short-term and relatively long-term IOP dynamics in eyes with POAG. For this, we reviewed our medical records and enrolled POAG patients whose IOP were measured with GAT at least three times and who underwent DRPT during the follow-up period. We found that IOP change during DRPT or IOP after DRPT were significantly associated with IOP fluctuation and IOP max during the follow-up period after adjustment for other clinical parameters, even in eyes with POAG.

We found that IOP increased 6.13 ± 3.55 mmHg on average in response to DRPT even in eyes with POAG. There has been limited investigation into IOP changes during DRPT in eyes with an open angle, possibly because DRPT was initially developed for detecting eyes with angle closure. Friedman et al. applied DRPT not only to primary angle closure suspects (PACS) but also to healthy open-angle eyes. They found that both PACS and open-angle eyes showed IOP increases in response to DRPT (4.25 ± 2.99 mmHg and 5.23 ± 2.77 mmHg, respectively), indicating that it is difficult to distinguish them based on their DRPT results [17]. While a direct comparison between their results and ours is not feasible due to differences in the presence of glaucoma, the time the subjects spent in the prone position duration (15 m vs. 60 m), axial length (23.5 ± 1.04 mm vs. 25.94 ± 1.71 mm), and baseline IOP (15.17 ± 3.08 mmHg vs. 12.65 ± 2.18 mmHg), we support the idea that IOP increase is not exclusive to eyes with angle closure.

We observed that the degree of IOP elevation varied to some extent in eyes with open-angle glaucoma, as indicated by the SD of 3.55 mmHg. Although IOP elevation due to postural changes has been well-documented for an extended period [15, 16, 25], the mechanisms underlying elevated IOP are intricate, and much remains to be discovered. Nelson et al. proposed that IOP changes can be accounted for by hydrostatic forces, in conjunction with an autoregulatory component, although the prone position was not used in their study [26]. Hypertension has been reported to correlate with a more significant increase in postural IOP [27]. Additionally, choroidal vascular congestion resulting from postural variation may be associated with postural IOP changes [14]. Various factors could influence the impact of DRPT on IOP, warranting further investigation [4, 6].

This study revealed that IOP after DRPT was associated with IOP max during follow-up, even when adjusted for various clinical parameters. Though it is clinically important to ensure that peak IOP during follow-up does not exceed the target IOP, there are not many reports on its clinical significance. De Moraes et al. investigated factors contributing to the progression of visual field defects in treated glaucoma and showed that peak IOP during follow-up was a contributor [12]. Indeed, they argued that their results were supported by randomized controlled trials, which suggests that progression is less likely when the peak IOP is 18 mmHg or below [28]. Furthermore, Susanna et al. demonstrated that peak IOP during water-drinking provocation, which is a short-term provocation lasting less than one hour, is also associated with the severity and progression of glaucomatous visual field defects [29, 30]. Although our clinical background and follow-up observation period differ from De Moraes et al., and our provocation method differs from Susanna et al., comprehensive consideration of our results and previous reports suggests that peak IOP during follow-up can be somewhat predicted by peak IOP during the provocation test, which in turn could potentially serve as an indicator of the progression of visual field defects. In this study, due to our focus on the correlation between short-term and relatively long-term IOP, and from the perspective of minimizing any changes in the eye condition due to eyedrop effects and long-term follow-up observation, we limited the follow-up period to less than 2 years. Therefore, there were few patients for whom a reliable mean deviation slope could be calculated, and it can be said that the question of whether these are truly related is a subject for future research.

We also found an association between IOP change in DRPT and IOP fluctuation during follow-up, indicating a link between short-term and relatively long-term IOP fluctuation. Even if average IOP is within the normal range, a larger fluctuation in IOP during follow-up may be a risk factor for faster glaucoma progression [7, 10]. To detect fluctuation in IOP, multiple outpatient visits are necessary over a certain period. Therefore, it would be clinically useful if we could predict long-term IOP fluctuations with shorter-term tests, particularly within one day. Tojo et al. reported that IOP fluctuation in a day measured with a contact lens sensor correlated with long-term IOP fluctuation [31]. Fogagnolo et al. measured inpatients’ IOP every four hours over a 24-h period and also collected data on IOP fluctuation during a two-year follow-up period. They reported that both IOP fluctuation during a 24-h hospitalization and during the follow-up contributed to glaucomatous VF defect progression [32]. We believe our method is superior in a clinical setting, as it does not require any special devices, can be performed as an outpatient procedure, and can be completed in one hour. However, as mentioned earlier, we were unable to analyze the relationship between the progression of VF defects and IOP dynamics in DRPT. Therefore, in the future, we are eager to study the relationship between the progression of VF defects and IOP change in DRPT.

Our study has several limitations. It was retrospective and conducted at one institute, which could have introduced subject bias. In Japan, 70% of glaucoma cases are normal-tension glaucoma (NTG) [33], and at our clinic specifically, NTG comprises about 60% of broadly defined POAG cases. In addition, glaucoma patients with IOP over 21 mmHg account for only 5% of all POAG patients. Our patient population predominantly showed progressive visual-field defects despite having normal IOP [34], and cases of typical high-tension glaucoma were relatively rare, indicating bias in this study. However, it can also be argued that these very patients are the group that needs thorough investigation with provocation tests like DRPT to determine if IOP truly does not play a role in the pathophysiology of their illness. While we admit that the results may not be applicable to all glaucoma cases, we believe that they hold significant value for this subtype of glaucoma. In addition, we excluded many patients from the group that underwent DRPT, mainly because of a history of medical interventions, such as surgery, or eyedrop add-ons during follow-up, as shown in Fig. 1. This was essential to exclude the effect of anti-glaucoma treatment on IOP dynamics; however, this selection process could also have led to bias. Thus, we additionally analyzed data from all 1023 eyes of 556 subjects for which at least 3 measurements of IOP were available and confirmed that the main results did not change (i.e., IOP max was significantly associated with IOP after DRPT and IOP fluctuation was significantly associated with IOP change during DRPT [multivariable linear mixed-effects model: *p* < 0.05]). Therefore, we do not consider that our selection process led to notable bias in our results. Second, we could not eliminate the effects of topical anti-glaucoma medications on our results. Nevertheless, it has been reported that the most common glaucoma eyedrop components, prostaglandin analogs and beta-blockers, have no impact on IOP changes due to postural shifts [35, 36]. Furthermore, we incorporated the number of eyedrops as an explanatory variable in the statistical models for Tables 2 and 3, and we therefore believe that the main results in this study are relatively solid. The third limitation is related to the duration of DRPT. We conducted DRPT for 60 min, following the method originally reported by Harris et al. [16]. However, recently, there have been several attempts to conduct DRPT in even shorter periods of time [17]. In our study, we did not change the body position of the subjects or measure IOP in the middle of the test; we measured IOP with GAT only before and after the provocation and only in the sitting position. In the future, we would like to explore whether we can further shorten the duration of DRPT for an even easier clinical application of the test. The fourth limitation is the effect of blood pressure (BP). Zhao et al. conducted a meta-analysis and reported that hypertension was associated with increased IOP [37]. While lying down, BP might rise abnormally in some patients [38]. In addition, systemic changes induced by hypertension could be related to the variability of BP [39, 40]. In the current study, we did not measure BP before and during DRPT. However, a 40 mmHg increase in blood pressure due to HT is required for IOP to rise by 1 mmHg, based on the calculation by Zhao et al. In addition, we measured IOP with GAT after the patients returned to the sitting position. To consider the effect of a history of HT based on our available data, we reviewed the patient forms and found that 11 patients (19 eyes) had a history of HT. We analyzed the effect of a history of HT on IOP after DRPT and IOP change during DRPT with linear mixed-effects models. We found that HT was not associated with IOP after DRPT (β = -0.15; *p* = 0.305) or IOP change during DRPT (β = -0.22; *p* = 0.141). In addition, we added HT as an explanatory variable to the linear mixed-effects models in Tables 2 and 3. IOP max was significantly associated with IOP after DRPT (β = 0.38; *p* < 0.001). IOP fluctuation was significantly associated with IOP change in DRPT (β = 0.28; *p* = 0.011). These associations did not change after adjusting for HT. Taken these findings together, we consider that HT did not have a great impact on the current study.

In conclusion, we found a relationship in eyes with POAG between IOP dynamics during the one-hour DRPT and IOP fluctuations during a comparatively longer period of follow-up. Thus, the DRPT allows clinicians to predict IOP fluctuation during follow-up to some extent.
